# Suitability of Piperacillin–Tazobactam for Treating Dogs Infected with Extended-Spectrum β-Lactamase-Producing Enterobacterales: Pharmacokinetic and Pharmacodynamic Analysis

**DOI:** 10.3390/antibiotics14050425

**Published:** 2025-04-23

**Authors:** Kazuki Harada, Hyo Harada, Yuka Kanao, Mizuki Kusumoto

**Affiliations:** 1 Laboratory of Veterinary Internal Medicine, Tottori University, Minami 4-101, Koyama-Cho, Tottori-shi 680-8550, Tottori, Japan; 2 Joint Graduate School of Veterinary Sciences, Tottori University, Minami 4-101, Koyama-Cho, Tottori-shi 680-8550, Tottori, Japan

**Keywords:** dogs, extended-spectrum β-lactamase-producing Enterobacterales, pharmacokinetic–pharmacodynamic approach, piperacillin–tazobactam

## Abstract

**Background/Objectives**: Piperacillin–tazobactam (TZP) is a potential alternative to carbapenems for the treatment of dogs infected with extended-spectrum β-lactamase-producing Enterobacterales (ESBL-E), but its efficacy remains unestablished. In this study, pharmacokinetic–pharmacodynamic (PK/PD) analysis was performed to estimate the clinical efficacy of TZP against ESBL-E infections in dogs. **Methods**: We determined the minimum inhibitory concentrations (MICs) of TZP in canine ESBL-E isolates, including *Escherichia coli* (*n* = 62), *Klebsiella pneumoniae* (*n* = 89), and *Enterobacter cloacae* (*n* = 31), using agar dilution. Monte Carlo Simulation (MCS) was performed to estimate the probability of target attainment (PTA) based on the PK/PD characteristics of TZP. **Results**: The MICs that can inhibit the growth of 90% of the isolates for the three bacterial species were determined as 16/4 µg/mL. MCS analysis revealed that the piperacillin PK/PD cutoff values (highest MICs with a PTA ≥90%) were ≤0.031, ≤0.5, and ≤2 μg/mL at a bolus dose of 50 mg/kg TZP (44.4 mg/kg piperacillin) every 12, 8, and 6 h (q12h, q8h, and q6h), respectively. The cumulative fractions of response were ≤90% based on the MIC distribution of ESBL-producing *E. coli*, *K. pneumoniae*, and *E. cloacae* isolates from dogs: 1.60, 0.48, and 0.15% at q12h; 32.56, 14.57, and 9.65% at q8h; and 74.51, 45.85, and 43.92% at q6h, respectively. **Conclusions**: We believe that TZP is not recommended for the treatment of canine ESBL-E infections, except for cases with a lower MIC than the PK/PD cutoff values determined in this study.

## 1. Introduction

**Figure 1:**
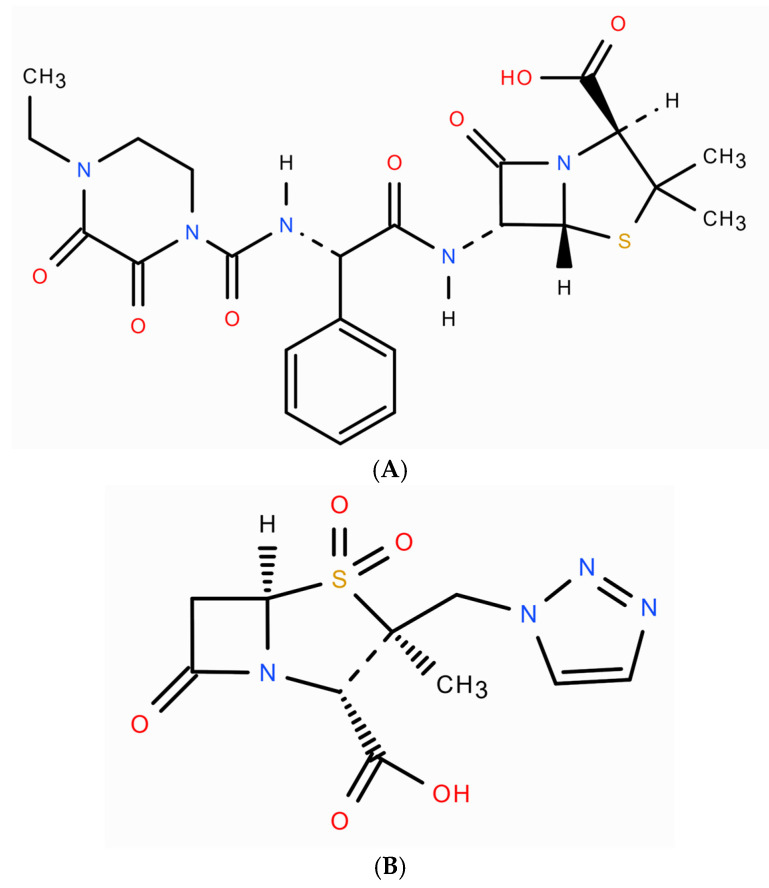
The chemical structures of (**A**) piperacillin and (**B**) tazobactam.

Extended-spectrum β-lactamase-producing Enterobacterales (ESBL-E) are a significant concern in veterinary medicine and human healthcare worldwide [1,2]. We previously conducted a pharmacokinetic (PK)–pharmacodynamic (PD) analysis on cephamycins to determine the potential of these antibiotics as an alternative treatment option for canine ESBL-E infections [3,4,5]. However, these antibiotics are only available in some Asian countries [6,7], and there is a paucity of research on other classes of antimicrobials. Piperacillin–tazobactam (TZP) is a β-lactam–β-lactamase inhibitor combination that exerts a broad spectrum of activity against Gram-negative bacteria, Gram-positive bacteria, and anaerobes [8]. Figure 1 presents the chemical structures of piperacillin and tazobactam. Moreover, this drug is a potential alternative to carbapenems for the treatment of human ESBL-E infections [9,10,11]. However, whether TZP and carbapenems elicit equivalent outcomes for ESBL-E infections in humans remains unclear because of inconsistent findings on TZP treatment across clinical studies [10,11]. Under these circumstances, Tamma and Rodriguez-Bano [11] recommended that TZP should be prescribed only for low- to moderate-severity infections, urinary or biliary tract infections, and infections of highly piperacillin-susceptible ESBL-E. Although TZP is widely used for companion animals [12], its efficacy in dogs with ESBL-E infections remains unestablished.

In this study, we assessed the in vitro efficacy of TZP on representative ESBL-E species (i.e., *Klebsiella pneumoniae* and *Enterobacter cloacae*), as well as *Escherichia coli* [13]. Furthermore, we performed Monte Carlo Simulation (MCS) analysis to determine canine-specific nonclinical PK/PD cutoff values for TZP and to estimate the potential efficacies of different TZP dosing regimens for canine ESBL-E infections.

## 2. Results and Discussion

The distribution of the minimum inhibitory concentrations (MICs) of TZP in ESBL-producing isolates of *K. pneumoniae* and *E. cloacae* from dogs is shown in Table 1. The MIC50 and MIC90 values (MICs that can inhibit the growth of 50% and 90% of the isolates, respectively) for the two species of ESBL-E were almost identical to those of *E. coli*. However, relatively lower TZP susceptibility rates were observed in the two bacterial species compared with *E. coli*, although no statistically significant differences were observed among the three species in multiple comparisons using Marascuilo’s method (*p* > 0.05). TZP can be hydrolyzed by AmpC enzymes [14]; therefore, we investigated the relationship between TZP MICs and plasmidic AmpC-type β-lactamase (PABL) in the three species. As for PABL-positive isolates, 2/5 *E. coli*, 7/8 *K. pneumoniae*, and 3/7 *E. cloacae* were classified as susceptible, and thereby, a strong association was not found between PABL and TZP susceptibility. Several reports have described resistance mechanisms other than that of PABL. Hubbard et al. [15] identified TZP resistance due to the IS*26*-mediated amplification of *bla*_TEM-1B_ in an *E. coli* isolate. Han et al. [16] demonstrated that *K. pneumoniae* isolates hyperproducing *bla*_SHV-1_ or *bla*_SHV-11_ β-lactamases exhibited TZP resistance. Guérin et al. [17] reported that AmpC derepression, mainly resulting from *ampD* mutations, led to an eightfold increase in the TZP MIC in *E. cloacae*. Although these mechanisms were not identified in our study, such species-specific TZP resistance mechanisms should be considered when administering TZP to dogs.

**Figure 2:**
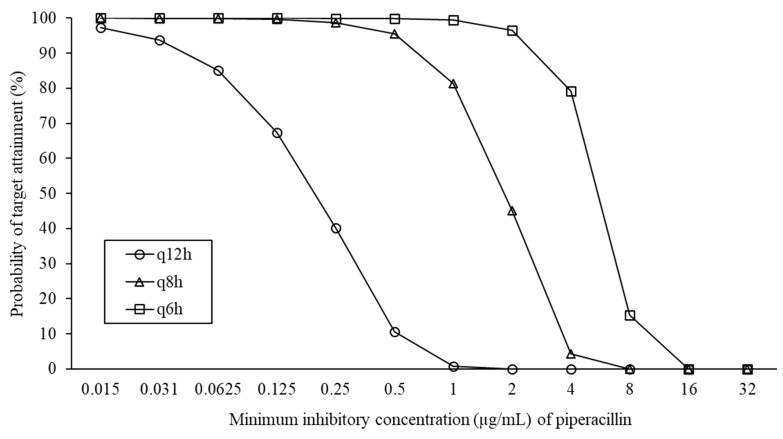
Probability of target attainment (%) at each minimum inhibitory concentration of piperacillin following dosage of 50 mg/kg of piperacillin–tazobactam (44.4 mg/kg piperacillin). Broken line indicates 90% probability of target attainment. q12h, q8h, and q6h mean dosing every 12, 8, and 6 h.

In human medicine, PK/PD analysis for TZP has been performed using the PK data of piperacillin [18,19,20]. Based on such precedence, we first attempted to conduct a PK/PD analysis for TZP in dogs. Figure 2 shows the probability of target attainment (PTA) results at each MIC of piperacillin when TZP is administered at the respective dosing regimens. Notably, an increase in the MIC is generally associated with a decreased PTA. However, when the drug was administered at dosing intervals of q12h (every 12 h), q8h, and q6h, the PTAs at MIC values of 0.031, 0.5, and 2 μg/mL were 93.78, 95.52, and 96.62%, respectively. The results suggest that shortening the dosing interval can help maintain a sufficient PTA even at higher MICs. In contrast, at MIC values above this range, the PTA dropped below 90%, indicating insufficient target attainment. Based on these results, the nonclinical PK/PD cutoff values for a bolus dose of 50 mg/kg TZP administered at q12h, q8h, and q6h were established as ≤0.031, ≤0.5, and ≤2 μg/mL, respectively. These values were lower than the TZP MIC50 and MIC90 in ESBL-E obtained in this study, implying that the 50 mg/kg TZP regimens are insufficient for inhibiting the growth of most ESBL-E isolates from dogs. Furthermore, these values were lower than the Clinical and Laboratory Standards Institute (CLSI) susceptibility breakpoint of ≤8/4 μg/mL for dogs [21], which was extrapolated from that for humans [22]. One possible reason for the lower PK/PD breakpoints is that the TZP dose used for dogs is smaller than that for humans, which is 3.375–4.5 g (56.25–75 mg/kg for adults weighing 60 kg) per dose q6h [8]. As for the regimens evaluated in this study, a dose of 50 mg/kg has been widely authorized in dogs [23]. Furthermore, a previous study reported that the maximum nontoxic dose of TZP in dogs is 200 mg/kg/day, which corresponds to 50 mg/kg q6h [24]. Thus, we believe that the TZP regimens in this study are acceptable from the viewpoints of safety and efficacy. These findings indicate the need to establish a lower value for canine-specific TZP breakpoints.

Furthermore, we calculated the cumulative fractions of response (CFRs) of TZP based on the MIC distribution of ESBL-E isolated from dogs (Table 2) and found that none of the TZP regimens evaluated in this study could achieve a CFR ≥ 90%, although a relatively higher CFR was observed in *E. coli* compared with *K. pneumoniae* and *E. cloacae*. Therefore, based on the results of the PK/PD analysis, none of the regimens were optimal for the treatment of dogs with ESBL-E infections. In human medicine, the continuous infusion of β-lactams, including TZP, may result in more favorable outcomes than intermittent administration [25]. In the present study, we tentatively calculated the steady-state serum TZP concentration through 24 h continuous infusion. Assuming 200 mg/kg TZP (177.78 mg/kg piperacillin) was continuously infused for 24 h, the piperacillin K0 would be 7.41 mg/kg/h. Thus, the mean piperacillin Css would be 29.63 mg/L (24.27 mg/L piperacillin fCss). This value is higher than the piperacillin concentrations of TZP MIC90 for ESBL-E isolates from dogs (i.e., 16 mg/L); however, it is unlikely to attain Css/MIC > 5, which is the target value of continuous β-lactam infusion to prevent the occurrence of microbiological failure, as proposed by Gatti et al. [26]. Therefore, future studies are needed to validate whether the continuous infusion of TZP provides greater efficacy than intermittent administration in dogs.

**Table 1:** Distribution of minimum inhibitory concentration (MIC) of piperacillin–tazobactam (TZP) in extended-spectrum β-lactamase-producing Enterobacterales isolates from dogs.

Bacterial Species ^1^	No. of Isolates with TZP MIC (µg/mL) ^1,2^										MIC50 ^4^ (µg/mL)	MIC90 ^5^ (µg/mL)	No. of Susceptible Isolates (%)
	1/4	2/4	4/4	8/4	16/4	32/4	64/4	128/4	256/4	512/4			
*E. coli* [*n* = 62 (5)] ^3^	6	20	26 (1)	4 (1)	3 (2)	1	0	1 (1)	1	0	4/4	16/4	56 (90.3)
*K. pneumoniae* [*n* = 89 (8)]	2	9 (1)	26 (2)	31 (4)	15	3 (1)	0	1	1	1	8/4	16/4	68 (76.4)
*E. cloacae* [*n* = 31 (7)]	0	1	13 (1)	8 (2)	6 (2)	2 (1)	0	0	1 (1)	0	8/4	16/4	22 (71.0)

^1^ The number of isolates in parentheses indicates those harboring plasmidic AmpC β-lactamases, as identified in previous studies [13,27]. ^2^ The vertical line indicates the susceptible breakpoint established by the Clinical and Laboratory Standards Institute [21,22]. ^3^ TZP MICs in *Escherichia coli* were determined in a previous study [13]. ^4^ The MIC that can inhibit the growth of 50% of the isolates. ^5^ The MIC that can inhibit the growth of 90% of the isolates.

**Table 2:** Cumulative fraction of response in piperacillin–tazobactam regimens against MIC distribution of ESBL-producing Enterobacterales isolated from dogs.

Dosing Intervals	Cumulative Fraction of Response (%)		
	*Escherichia coli*	*Klebsiella pneumoniae*	*Enterobacter cloacae*
q12h	1.60	0.48	0.15
q8h	32.56	14.57	9.65
q6h	74.51	45.85	43.92

Several limitations should be acknowledged in this study. Firstly, the number of ESBL-E isolates was relatively small, which may reduce the reliability of the derived PD parameters. Secondly, a time–kill study—an established method for evaluating antimicrobial efficacy—was not performed. However, López-Cerero et al. [28] investigated the efficacy of TZP against ESBL-producing *E. coli* strains using a time–kill study. They reported that TZP at concentrations of 2–8 × MIC resulted in a reduction of less than 1 log_10_ colony-forming unit (CFU)/mL at high inoculum levels after 8 h, whereas it achieved a reduction of more than 3 log_10_ CFU/mL at standard inoculum levels. Such inoculum effects of TZP should be considered, alongside the present findings, when using this drug in dogs.

## 3. Materials and Methods

We determined the MICs of TZP for 120 ESBL-E isolates from dogs, consisting of *K. pneumoniae* (*n* = 89) and *E. cloacae* (*n* = 31), which were previously obtained from collections sent to commercial labs for clinical purposes [28]. The production of ESBL, together with PABL, was phenotypically identified using commercial disk sets (ABL and ESBL Detection Set, MAST Group Ltd., Merseyside, UK) in a previous study [28]. The MICs were determined using the agar dilution method, as recommended by CLSI guidelines [21]. A series of Mueller Hinton agar plates containing a twofold serial dilution of piperacillin (0.031–512 μg/mL) combined with 4 μg/mL of tazobactam was prepared. The bacterial inoculum was prepared by suspending several colonies in sterilized saline to achieve turbidity equivalent to a 0.5 McFarland standard. The bacterial suspension was spot-inoculated onto the surface of each plate with approximately 10^4^ CFU per spot using an MIT-60P Microplanter (Sakuma Seisakusho, Co., Ltd., Tokyo, Japan). After incubation at 35 °C for 18 h, the lowest concentration of the drug that completely inhibited visible bacterial growth was recorded as the MIC. Susceptibility was interpreted according to the CLSI breakpoint (susceptible: ≤8/4 μg/mL) [21]. The *E. coli* ATCC 25922 and *Pseudomonas aeruginosa* ATCC 27853 strains were used as quality control organisms to ensure the reliability of the MIC data.

MCS was performed using Oracle Crystal Ball version 11.1.2.4.850 (Kozo Keikaku Engineering Inc., Tokyo, Japan) to estimate the PTA based on the PK and PD characteristics of TZP. In this study, 5000 drug–serum concentration–time profiles were developed based on a one-compartment model using PK parameters when administered with tazobactam (i.e., total body clearance [CL: 0.25 ± 0.03 L/h/kg] and volume of distribution [Vd: 0.23 ± 0.04 L/kg]) derived from a study by Zaghloul et al. [29]. We adopted 18.1% as the canine-specific serum protein-binding rate of TZP [30]. Furthermore, we used the time taken by the unbound drug concentration to exceed the MIC (fTAM) ≥50% as the PK/PD target value for the efficacy of TZP, as reported previously [18,19,20]. The fTAM (%) was calculated using the following equation [31,32]:

fTAM (%) = ln [(dose × f) ÷ (Vd × MIC)] × (Vd ÷ CL) × (100 ÷ DI),

where f is the fraction of unbound drug, and DI is the dosing interval (h). Furthermore, Vd and CL were presumed to be log-normally distributed with standard deviations derived from a previous report [29].

The nonclinical PK/PD cutoff values of TZP were calculated as the highest MIC that achieved a PTA ≥ 90% [20,33] when administered at 50 mg/kg (44.4 mg/kg piperacillin, based on an 8:1 ratio of piperacillin–tazobactam) at q12h, q8h, and q6h. The CFR was determined using the MIC distribution of TZP in ESBL-E isolated from dogs. A regimen was considered optimal at a CFR ≥ 90% [20,34].

To assess the feasibility of continuous TZP infusion, the steady-state serum concentration (Css) was calculated using the following equation [35]:

Css = K0/CL,

where K0 is the infusion rate.

## 4. Conclusions

We established canine-specific PK/PD cutoff values for TZP regimens of a bolus dose of 50 mg/kg at q12 h, q8 h, and q6h based on traditional PK/PD analysis and determined CFRs based on the MIC distribution of ESBL-E isolated from dogs. The PK/PD cutoff values determined in this study could be increased by reducing the dose interval; however, all these values were lower than the CLSI breakpoints. Furthermore, the present TZP regimens likely have low CFRs for dogs infected with ESBL-producing *E. coli*, *K. pneumoniae*, and *E. cloacae*. TZP has been listed as a critically important antimicrobial by the World Health Organization [36] and should, therefore, be used prudently in veterinary medicine. From these points of view, TZP is not recommended for the treatment of canine ESBL-E infections, except for cases with a lower MIC than the PK/PD cutoff values determined in this study.

## Data Availability

The data presented in this study are available from the corresponding author by reasonable request.
